# Prevention and Treatment of Peritoneal Metastases from Gastric Cancer

**DOI:** 10.3390/jcm10091899

**Published:** 2021-04-28

**Authors:** Paul H. Sugarbaker

**Affiliations:** Washington (D.C.) Cancer Institute at Washington Hospital Center, Washington, DC 20007, USA; Paul.Sugarbaker@outlook.com

Common knowledge would suggest that regional treatments for peritoneal metastases from gastric cancer would not be successful. The natural history of this disease shows a strong propensity to widely disseminate through lymphatic and hematogenous routes of metastases in addition to intracoelomic spread. With this widespread pattern of spread, the success that is being reported in the management of gastric cancer with cytoreductive surgery and perioperative chemotherapy may be unexpected. One important consideration is the large number of patients in whom peritoneal dissemination of the disease is the first and most deadly pattern of cancer dissemination [1,2]. In other words, peritoneal metastases are very common in gastric cancer patients and when they occur the patient’s life expectancy is only a few months. It is the most aggressive pattern of gastric cancer dissemination.

First, I want to think about the prevention of peritoneal metastases. In this clinical situation, the target for perioperative regional chemotherapy, usually hyperthermic intraperitoneal chemotherapy (HIPEC), is one for which regional chemotherapy is successful. Surgery removes the primary disease and peritoneal dissemination is limited to free cancer cells. Established peritoneal implants that are vascularized or lodged in lymphatic organelles are not present. HIPEC has both the mechanical effects of removing large numbers of cancer cells and complete penetration of single cells or minute groups of cells by cancer chemotherapy. It is expected to be successful in this clinical situation.

In contrast, using perioperative chemotherapy to treat visible peritoneal implants presents a much greater challenge. For vascularized tumor nodules and cancer growing within the lymphatic organelles of the peritoneal surface, there is limited access of cancer chemotherapy into these organized tumor deposits. Additionally, capillary or lymphatic blood flow will rapidly remove the chemotherapy from contact with the cancer nodule and into the systemic circulation. Use of regional chemotherapy in addition to cytoreductive surgery in patients with established gastric cancer peritoneal metastases should have limited expectations for an improved outcome. More effective HIPEC or different and more effective perioperative treatments are required. It is possible that a treatment that will delay the progression of peritoneal metastases (the deadliest component of this disease) may show limited benefits [3,4].

A third use of regional chemotherapy is repeated instillations, sometimes referred to as normothermic intraperitoneal chemotherapy (NIPEC). These treatment plans have limited goals to eradicate, with multiple and repeated instillations of intraperitoneal chemotherapy, the peritoneal component of gastric cancer dissemination. This combined with some help with disease control from systemic chemotherapy may allow the “super responder” to undergo a complete resection of a large tumor mass as the multiple small but invasive tumor nodules on peritoneal surfaces have been controlled by NIPEC. “Conversion surgery” is great when it is possible, but at this point in time, only a small proportion of patients benefit [5,6].

In some respects, gastric cancer may be unique in terms of the management of peritoneal metastases. Despite its propensity to rapidly become a systemic disease process, interventions to control the peritoneal metastases component of this disease process can result in an improved outcome. The Special Issue on Advances in Peritoneal Carcinomatosis from Gastric Cancer edited by Gonzalez-Moreno and Ortega-Perez presents data showing success with a regional approach to selected patients with gastric malignancy. Congratulations to them in this timely effort to improve the outcome of peritoneal metastases from gastric cancer. 
